# A Georgia Doctor in New York

**Published:** 1886-12

**Authors:** K. P. Moore

**Affiliations:** New York


### A GEORGIA DOCTOR IN NEW YORK.

*Editors Atlanta Medical and Surgical Journal:*

According to promise I undertake to give you an occasional letter from this place.

A little more than a month's stay has already served to confirm what I had before learned, that New York, in medicine, as in every other department of life, is certainly "headquarters."

I find the schools all unusually full. The classes at University, and College of Physicians and Surgeons, each number over five hundred, while at Bellevue there are between four and five hundred. The class at the polyclinic has over sixty, while the post-graduate is not quite so large. Including the visiting physicians who are here looking around, I think it would be safe to say that there are now in the city at least two thousand students of medicine. I mean in the regular school. At no time in the history of New York has there existed such a thirst for teaching as now. Besides the legitimate faculties and their adjuncts, numberless private classes and quizes are offering their services in every department, so that one finds quite a plethora of opportunities presented.

To one who has been busily confined to active work for eighteen years, it is indeed pleasing to note the real advancement made in almost every department of medical science. The great number of teachers and aspirants for promotion necessarily created quite a rivalry, and hence thorough and careful exploration and search after something new; this, together with the abundant clinical material offered by this large city, has enabled the profession here to carry our banner full abreast with the other sciences in the spirit and progress of the age, if indeed it is not far in advance. Though in some instances one feels that a bit too much of humanity may be laid upon the altars of science, and while the profession may reap valuable information, it may sometimes happen that the life of the patient is the price paid for it. Laparotomy is almost daily made for diagnostic purposes, and I would suggest that it may be indulged in too frequently, though Listerism has reduced the rate of mortality to a degree that is truly wonderful.

It may appear a little presumptuous in me to criticize anything I see here, but in my judgment gynæcological surgery is running into extremes in certain directions. Tait's and Alexander's operations are too frequently indulged in, and, as remarked by an eminent surgeon of this city a few days ago, I think it is time that the profession was rising up and calling a halt. The tendency of the profession has always been to follow the fashion, and, like the devotees of every fashion, to go into extremes. Besides the danger to the patients (which I grant is small), there is danger of bringing a good remedy into disrepute. Already the operation is derisively spoken of as "spaying," etc. While I feel proud of our Battey as the pioneer in this hitherto unexplored domain of surgery, and believe that the honors ought to rest upon his brow, yet I fear that his operation, and that as modified by Tait, is in danger of suffering at the hands of reckless operators, who seem to have more thirst for fame than regard for the preservation of those organs and functions which go to make a pure woman, fitted in every particular to occupy her proper sphere in life. A good many of the more conservative men here are of my opinion and condemn the reckless manner of operating. I was amused some days ago at an expression of a learned professor in one of his lectures. He had occasion to speak of the prevalence of these various operations and referred to the obstetricians and gynæcologists as "our bloody brethren." While I had never before heard that title conferred upon them, yet I rather think that it is one justly merited, and therefore would not desire to deprive them of it, though after all we must admire, if we do not approve, their pluck and energy, and I mete out to them their full meed of praise and congratulations.

It is in this department of surgery that Listerism has enjoyed its greatest triumphs, and now it has come to be true that almost any operation in surgery, under the benign influence of antiseptics, has been shorn of its dangers, and we now undertake the most dangerous operation with perfect confidence, so far as the inflammatory consequences incident to the operation are concerned.

Every operation here, whether great or small, is done antiseptically, and whether the operation is for the removal of an ingrowing toe-nail, or the exsection of a portion of the alimentary canal, the operator would almost be held criminal if he did not use antiseptics. By the way, this reminds me to speak of an operation at Mt. Sinai Hospital some days ago, by Dr. J. A. Wyeth, on a woman sixty-five years of age. He removed two or three inches of the small intestines which had been strangulated in a hernia. The temperature never went above 100, and only for a few hours did it ever reach that point; usually it did not go above 99. Five days after the operation she had a natural move on the bowels, and at this writing, now about ten days, the patient is about well and "Richard is himself again." Dr. Wyeth, I understand, has several times made exsections of portions of the alimentary canal and each time successfully.

In all operations here the rule is never to disturb the dressing from the time it is put on until it is well. The temperature is the guide, and unless it goes up and persists, or unless the symptoms should indicate secondary hemorrhage, or something of this sort, the dressing is never disturbed until due time has elapsed for perfect union and recovery. I have seen a great many dressings removed after operations of almost every variety, including bone and serous tissues, and I cannot now recall a single instance in which there was a drop of pus.

Our old friend, Dr. Sayre, is still as enthusiastic over the nervous reflexes produced by congenital phymosis and adherent prepuce as ever, and I have seen him wonderfully sustained by the actual results of his operations. Several cases, which I have seen through his courtesy, would be well worth reporting, but as my letter has already been rather drawn out, I will mention only one, which will illustrate three or four others now under observation.

A boy eleven years of age was operated on two weeks ago before the class at Bellevue. There was inco-ordination and rigidity of the whole muscular system. The larger tendons were all rigidly contracted and fixed. The joints were all flexed and immovable. His legs were crossed and perfectly fixed, giving a beautiful illustration of the scissors' legs. His arms were tightly flexed and crossed over the breast, while his hands and fingers were closed and drawn; he had internal strabismus of the left eye; the muscles of his tongue suffered in the general inco-ordination so that he could not articulate, and he presented altogether a sad picture. These symptoms had existed with gradually increasing intensity almost from his cradle, and his parents thought he was an idiot. Besides this operation of circumcision, Dr. Sayre thought best, in view of the long existence of the rigidity of tendons and muscles, to practice tenotomy, and hence cut both tendo-achiles and the aductors of the thighs on both sides. He was then placed in bed with the legs abducted and tied to either side of the bed and the usual club-foot dressing upon the feet. I had the pleasure of visiting the patient with Dr. Sayre just two weeks from the day of operation and saw the dressings taken down. The tendons had all united perfectly, his legs did not cross at all, he could flex and extend the feet at will, not perfectly of course, and when stood upon his feet could make decided efforts at walking, a thing he had probably never done before ; he could talk first rate, and so far from being idiotic he struck me as being unusually bright and intelligent for one of his opportunities. He is now beginning to receive systematic education of his muscles by massage, flexion and extension and electricity, and I have no doubt will in a short time be made a very respectable boy. His legs are still smartly drawn and the doctor thinks he may have to cut the tendons under the knees. I could but rejoice to see such a revolution effected by our art, and it was pleasing to see the new hope which now begins to dawn upon the heart of this sad mother, to say nothing of the bright daylight of a useful life which seems now to be rising out of one hitherto doomed to utter helplessness and misery. The poor boy himself begins to appreciate the situation, and the happy sparkle of his eye as he talked to the doctor told a tale more pleasing than words. Space forbids the mention of the other cases, but they were all sufferers of great nervous reflex disturbances.

More anon. K. P. MOORE.

*New York, November, 1886.*

